# Supporting cardiac patient physical activity: a brief health psychological intervention

**DOI:** 10.1007/s00508-016-0968-y

**Published:** 2016-03-07

**Authors:** Marion Platter, Markus Hofer, Cornelia Hölzl, Alexandra Huber, Daniela Renn, Dave Webb, Stefan Höfer

**Affiliations:** 1 Department of Medical Psychology, Medical University of Innsbruck, Schöpfstrasse 23a, 6020 Innsbruck, Austria; 2 Faculty for Psychology and Sports Science, University of Innsbruck, Innsbruck, Austria; 3 Department of Cardiology, University Hospital Innsbruck, Innsbruck, Austria; 4 Department of Nutrition, University Hospital Innsbruck, Innsbruck, Austria; 5 University of Western Australia, Perth, Australia

**Keywords:** Coronary artery disease, Physical activity, Health action process approach, Health psychology, Follow-up study

## Abstract

**Background:**

One of the most important risk factors for coronary artery disease is physical inactivity. Health psychological research demonstrates the importance of planning for behaviour change success. Consequently, a health action process approach (HAPA) model-based design to support the uptake of physical activity was initiated for the first time in an acute cardiac ward.

**Methods:**

For impact evaluation, a control group (CG) and an intervention group (IG) of coronary artery disease patients were compared in a controlled longitudinal study. Baseline assessment included socio-demographic variables, intentions regarding physical activity, and actual physical activity prior to the coronary artery disease event. Follow-up data were collected 2 and 6 months after discharge.

**Results:**

In total, 193 patients participated in this controlled longitudinal study (63 ± 9 years; CG: *N* = 78; IG: *N* = 115). The IG reported a higher increase in physical activity (*p* < 0.05), intentions, and coping planning (*p* < 0.05), and also in action planning and control (*p* < 0.01) 2 months after discharge. Both CG and IG increased their physical activity 6 months after discharge to the point of no significant difference (*p* = 0.664).

**Conclusions:**

A HAPA model-based health psychological intervention on an acute cardiac ward is able to increase patients’ physical activity over the short term. However, integration of follow-up interventions (preferable in cardiac rehabilitation settings) would be necessary to support sustained physical activity.

## Introduction

Coronary artery disease is primarily a lifestyle-related disease and is the cause of the highest number of deaths in Western countries [1]. Several modifiable risk factors including smoking, hypertension, diabetes, hypercholesterolemia, lack of physical activity and psychosocial factors have been identified [2]. Although patients are often afflicted by two or more risk factors, multifactorial lifestyle interventions appear somewhat ineffective [3, 4]. Consequently, many studies have focussed on single risk factors. Lack of physical activity is one of the most important risk factors for myocardial infarction or coronary artery disease. Some studies reported protective effects of physical activity to a moderate extent [5].

Although knowledge about risk factors in respect to coronary artery disease is increasing, evidence regarding the predictors of healthy lifestyle uptake remains scant. One important exception is Ferrier et al.’s [6] study which revealed self-monitoring, enunciating specific goals, identifying barriers and developing plans as effective techniques for behavioural change post cardiac rehabilitation.

Beyond the above predictors, many behavioural studies present intention as an immediate and crucial antecedent to behaviour. However, Orbell and Sheeran [7] warn importantly that intention alone is not a sufficient predictor of behaviour change.

**Fig. 1:**
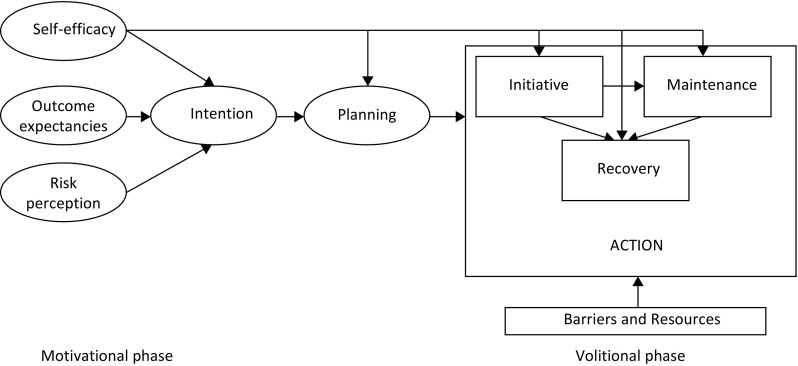
Health action process approach model [9]

In response, and specifically in a health context, the health action process approach (HAPA) [8, 9] model proposes that “planning” can bridge the intention-behaviour gap. Here, the HAPA model argues that health behaviour comprises two consecutive phases, the motivational and the volitional (Fig. 1). In the motivational phase, the formation of an intention to change health behaviour commences and is influenced by three factors: risk awareness, outcome expectancies and perceived self-efficacy. Once all three are sufficiently present, the intention is considered fully formed and the motivational phase complete. In the subsequent volitional phase the intended behaviour has to be initiated and then maintained over the longer term and even restarted in the event of setbacks.

Extensive evidence exists in a variety of settings supporting the usefulness of HAPA designed studies [10–16]. The study described in this paper extends this evidence by offering the first exploration of an evidence-based HAPA model intervention in an acute cardiac ward setting.

## Methods

### Recruitment and Intervention

A quasi-experimental study of patients with documented coronary artery disease and identified cardiac risk factors was carried out with ethics approval in a routine clinical practice setting at the Department of Cardiology, Medical University of Innsbruck.

Both a control group (CG) and an intervention group (IG) of patients who met the below criteria were recruited:

1. Diagnosis of coronary artery disease (acute coronary syndrome ACS or stable angina pectoris)
2. Sufficient knowledge of German
3. Ability to complete a set of surveys (i.e. no physical or mental impairments)
4. Physical agility (patients had to be able to reach the seminar room by foot)

The CG was recruited 2 months prior to implementing the intervention. Integration of the intervention in a routine cardiology setting prevented randomisation between the CG and IG.

Patients independently representing the CG and the IG signed an informed consent form prior to completing an identical survey (t0) at the time of recruitment. For the IG, prior baseline assessment was a requirement for group participation. Postal follow-up data were collected for both groups 2 (t1) and 6 months (t2) after discharge.

The intervention designed to fit within the clinical routine of an acute cardiac ward included direct patient participation in a 1-h group education session with a qualified and trained nurse. The intervention was carried out once a week for a 1-year time period and each IG patient participated only once.

The intervention comprised three parts: (a) information; (b) activation: developing personal action and coping plans; and (c) group discussion.

- *Information* about everyday physical activities for cardiac patients was presented and participants were invited to ask questions.
- Subsequently, patients were encouraged to develop their own *personal action and coping plans* concerning physical activity. Action plans are simple “when,” “where” and “how” plans to concretize the prospective healthy behaviour. A connection between situation (when, where) and behaviour (how) is built. An example of such an action plan could include: “I will run for 30 min along the river every evening.” Action planning helps to act in the intended way [17] and to initiate goal behaviour faster [6]. Coping planning serves as support to overcome potential obstacles and barriers that may frustrate planned behaviour by linking them to suitable coping strategies. For example: “It’s raining so I will go and swim for 30 min instead.”
- At the end of the session, patients were invited to *discuss* their plans within the group.

### Instruments

Data on socio-demographic and clinical variables were collected, and patient charts were reviewed for diagnosis and treatment. Self-reported risk factor items regarding level of physical activity, smoking, diabetes, hypertension, hypercholesterolemia and depression were asked. Depression was screened for with the two questions of the PHQ-2 questionnaire (a: “Over the past month, how often you have been bothered by feeling down, depressed or hopeless?” b: “Over the past month, how often have you been bothered by little interest or pleasure in doing things?”). A positive response to the two-item instrument has a sensitivity of 96 % (95 % confidence interval, 90–99 %) and a specificity of 57 % (95 % confidence interval, 53–62 %) in detecting depression [18]. Patients also provided information about potential confounding variables (participation in an inpatient or outpatient rehabilitation programme in the follow-up period, subjective assessment of satisfaction with physical activity and perceived impact of physical activity on health). Consistent with the HAPA model (Fig. 1), the survey included the following measures:

*General Self-Efficacy (GSE)*. To assess general perceived self-efficacy [19], the ten-item German version of the GSE using a 4-point response format “Not at all true,” “Hardly true,” “Moderately true” and “Exactly true” was included. A composite score is calculated with higher scores reflecting higher GSE. Prior studies revealed internal consistency ranging from 0.80 to 0.90 [20]. Criterion-related validity has been well documented [21].

*Measurement of intention, action and coping planning and action control*. Items measuring intention (seven items), action planning (five items), coping planning (five items) and action control (six items) for physical activity were included with slight wording modifications to fit the context of this study from those adopted by Sniehotta et al. [22] Example items included: I intend to be physically active several times a week (intention); I have made a detailed plan regarding when to be physically active (action planning); I have made a detailed plan regarding what to do if something interferes with my plans (coping planning); and, I have really tried hard to be physically active regularly (action control). Furthermore, the intention item “I intend to live healthy” was added to allow for a global appraisal. All items adopted a 4-point response format with 1 = not at all true to 4 = exactly true.

*Self-reported items on physical activity*. To control for the presence of potential confounding effects with respect to the patients’ subjective assessment of physical activity as well as the perceived impact of physical activity on health, two items derived from the German questionnaire on health behaviour (FEG) [23] were included. The two items: “How satisfied are you with your physical activity?” and “How does your physical activity impact your health?” were assessed on a 7-point scale from “extremely dissatisfied/negative” (− 3) to “extremely satisfied/positive” (3).

*Assessment of physical exercise*. An adaptation of the Kaiser Physical Activity Survey cf. Sniehotta et al. [15] for cardiac patients was included. Patients revealed how often and for how long per week (on average) they were active in each of five domains of physical activity: vigorous exercise (e.g. swimming, cycling), exercise to train muscle strength, fitness activities (e.g. gymnastics), game sports (e.g. football) and prescribed exercises (e.g. back exercises). For patients who did not act out any of the specified activities, the option “I did not act out any of the activities” was provided. The amount of physical activity per domain was calculated by multiplying days per week with minutes per session. To form a sum score, the five domains were aggregated.

### Statistical methods

Analysis commenced with an exploration of the data to ensure that no breeches regarding normal distribution were evident. The only measure revealing non-normality was physical activity. Consequently, based on the distribution, the Student’s *t* test or the Mann–Whitney U test was used to compare differences at baseline and changes over time between groups. The Wilcoxon signed-rank test was used to compare changes over time within groups. In addition, univariate and multivariate outliers were removed where appropriate. Frequencies and means (± SD) as well as median and quartiles (Q1; Q3) were used to describe clinical and socio-demographic variables (Table 1). Analysis of covariance was used to examine differences over time adjusted for baseline values and for significant baseline differences between groups. To determine and compare the effect size of the intervention and the CG Cohen’s *r* [*r* = z/√*N*] as an effect size for nonparametric data was calculated. Values: *r* > 0.1 represents a small effect, *r* > 0.3 a middle effect and *r* > 0.5 a large effect [24].

## Results

### Socio-demographic and clinical characteristics

A total of 121 CG and 153 IG patients met the aforementioned inclusion criteria. Of these, *N* = 78 (CG) and *N* = 115 (IG) were considered suitable for subsequent analysis. The mean age for the CG was 63 ± 9 years (IG: 64 ± 8.9 years); 59 % were men (IG: 70.4 %). There were 15 (19.5 %) patients with ACS in the CG and 14 (14.3 %) in the IG (*p* = 0.226). The rest of the patients were scheduled for elective coronary angiography (CAG). Different treatments at baseline were CAG followed by continued medical treatment (CG: *N* = 39; 51.3 %; IG: *N* = 54; 48.2 %); CAG followed by elective coronary artery bypass grafting (CG: *N* = 5; 6.6 %; IG: *N* = 10; 8.9 %), percutaneous transluminal coronary angioplasty (PTCA) (CG: *N* = 5; 6.6 %; IG: *N* = 1; 0.9 %), PTCA with stent implantation (CG: *N* = 26; 34.2 %; IG: *N* = 45; 40.2 %) and continued medical therapy only (CG: *N* = 1; 1.3 %; IG: *N* = 2; 1.8 %). No baseline differences for self-reported risk factors, intentions regarding the uptake of physical activity and GSE were found (Table 1).

In the CG at t1 *N* = 59 (75.6 %) and at t2 *N* = 56 (71.8 %) patients returned completed follow-up data. In the IG at t1 *N* = 87 patients (75.7 %); similarly at t2 *N* = 87 patients (75.7 %) participated.

**Table 1:** Patients’ characteristics at baseline (t0)

		CG (*N* = 78)	IG (*N =*115)	*p* value
		M ± SD, *N* (%), median (Q1/Q3)	M ± SD, *N* (%), median (Q1/Q3)	
Age		63.17 ± 9.1	63.83 ± 8.9	0.614^b^
Male		46 (59 %)	81 (70.4 %)	0.100^a^
BMI		27.59 ± 4.6	27.77 ± 4.3	0.793^b^
ACS		15 (19.5 %)	16 (14.3 %)	0.226^a^
Treatment	CAG + medical treatment	39 (51.3 %)	54 (48.2 %)	0.242^a^
	CAG + CABG	5 (6.6 %)	10 (8.9 %)	
	PTCA	5 (6.6 %)	1 (0.9 %)	
	PTCA + Stent	26 (34.2 %)	45 (40.2 %)	
	Continued medical treatment	1 (1.3 %)	2 (1.8 %)	
Self-reported risk factors	Smoking	9 (12.2 %)	19 (16.8 %)	0.383^a^
	PHQ-2 depression screening	15 (19.2 %)	17 (14.8 %)	0.454^a^
	Hypercholesterolemia	45 (62.5 %)	73 (67.6 %)	0.481^a^
	Diabetes	13 (18.1 %)	11 (11 %)	0.188^a^
	Hypertension	50 (67.6 %)	70 (65.4 %)	0.764^a^
Self-reported health (1 = excellent; 5 = bad)		3.16 ± 0.7	3.12 ± 0.7	0.765^b^
Self-reported health	Excellent	1 (1.3 %)	2 (1.8 %)	0.962^a^
	Very good	11 (14.3 %)	15 (13.3 %)	
	Good	41 (53.2 %)	65 (57.5 %)	
	Fair	23 (29.9 %)	29 (25.7 %)	
	Poor	1 (1.3 %)	2 (1.8 %)	
Educational background	< 10 years of education	62 (89.9 %)	82 (81.2 %)	0.257^a^
	10–15 years of education	6 (8.7 %)	14 (13.9 %)	
	> 15 years of education	1 (1.4 %)	5 (5 %)	
Occupation	Employed	19 (25.3 %)	27 (24.3 %)	0.993^a^
	Retired	50 (66.7 %)	75 (67.6 %)	
	Job seeking	1 (1.3 %)	2 (1.8 %)	
	Other	5 (6.7 %)	7 (6.3 %)	
General self-efficacy (1 = low; 4 = high)		3.23 ± 0.5	3.20 ± 0.6	0.746^b^
Intentions (1 = low; 4 = high)		2.95 ± 0.8	3.07 ± 0.7	0.294^b^
Satisfaction with physical activity (− 3 = low; +3 = high)		− 0.03 ± 2.1	0.2 ± 2	0.500^b^
Impact of physical activity on health (− 3 = low; +3 = high)		0.71 ± 1.8	0.94 ± 1.8	0.466^b^
Physical activity (min/week)		0 (0/135)	90 (0/225)	**0.047** ^**c**^
		98.95 ± 160.2	131.77 ± 150.1	

^a^Chi-squared test

^b^ *t* test for unpaired samples

^c^Mann–Whitney U test

### Confounding variables

No baseline differences in satisfaction with physical activity and perceived impact of physical activity on health were found between the CG and the IG (Table 1). Also, both groups reported a positive impact of physical activity on their health (CG: 0.71 ± 1.8, IG: 0.94 ± 1.8; *p* = 0.446). Furthermore, no difference in the frequency of participation in an alternative external rehabilitation programme in the follow-up period (t2) (*p* = 0.635) was revealed (CG: *N* = 10; 12.8 % and IG: *N* = 11; 9.6 %). Removing rehabilitation patients from the analyses did not alter the results.

### Patient’s knowledge regarding impact of deficient physical activity (t0)

No significant differences between the CG (2.34 ± 0.9) and the IG (2.17 ± 1.2) were revealed with regard to having knowledge that a lack of physical activity causes cardiovascular disease, diabetes, excess weight problems and mental diseases (*p* = 0.262) (Table 1).

### Between group differences in physical activity (t0)

A significant baseline difference in physical activity between the CG and IG was found (*p* = 0.047). Patients from the IG were overall 32.82 min/week more physically active than patients from the CG (Table 1). Minimum and maximum minutes of physical activity per week in CG and IG ranged from 0 to 720 (CG) and 0 to 630 (IG), respectively. This baseline difference was statistically controlled for in subsequent analysis.

### Between group differences on intention, planning items and action control (t1)

At month 2 (t1), IG patients reported higher intentions (CG: 3.02 ± 0.7, IG: 3.41 ± 1.6; *p* = 0.044), more action planning (CG: 2.75 ± 0.9, IG: 3.21 ± 0.8; *p* = 0.002), more coping planning (CG: 2.49 ± 0.8, IG: 2.77 ± 0.9; *p* = 0.033) and more action control (CG: 2.6 ± 0.9, IG: 3.1 ± 0.7; *p* = 0.001) than CG patients.

### Changes over time (t0–t1)

**Fig. 2:**
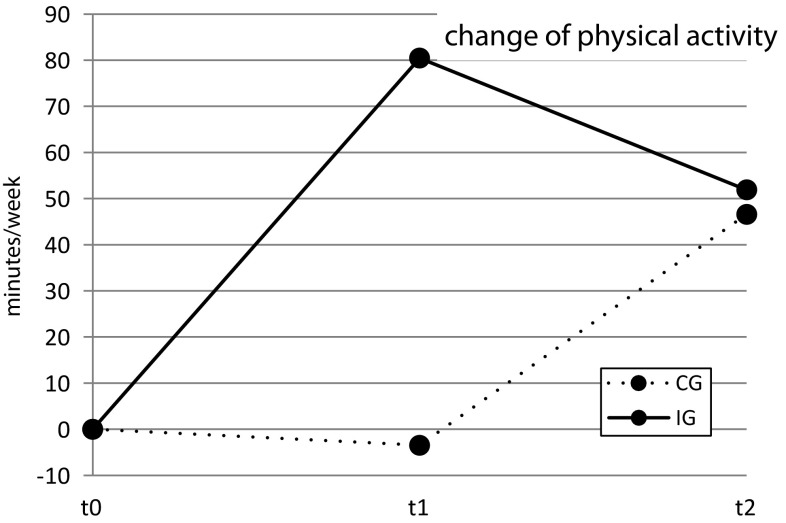
Change of physical activity over time (min/week) adjusted for baseline values and baseline differences; t0–t1 F(1, 120) = 8.31, *p* = 0.005; t0–t2 F(1, 122) = 0.18, *p* = 0.675

*Mean changes of physical activity (minutes/week)*. IG patients significantly increased their physical activity when compared to the CG (*p* = 0.021). At 2 months (t1), IG patients were 80.44 (± 213.2) minutes per week more physically active than they were at baseline (t0) (*p* = 0.001); in contrast, CG patients were 3.48 (± 207.4) minutes per week less physically active at month 2 (t1) than at baseline (t0) (*p* = 0.837) (Fig. 2). The IG showed a medium effect (*r* = 0.39), the CG none (*r* = 0.03).

*Mean changes in intention, physical activity satisfaction and impact of physical activity on health*. No differences in mean changes from baseline (t0) to month 2 (t1) between CG and IG in intention [F (1, 123) = 1.479, *p* = 0.226], physical activity satisfaction [F (1, 97) = 2.539, *p* = 0.114] and impact of physical activity on health [F (1, 94) = 2.539, *p* = 0.114] were found.

### Changes over time (t0–t2)

*Mean change in physical activity (minutes/week)*. Both CG and IG increased their physical activity from baseline (t0) to month 6 (t2) (CG: 46.58 ± 154.2, *p* = 0.008; IG: 51.89 ± 180.4, *p* = 0.012), though the difference was non-significant (*p* = 0.664) (Fig. 2). Both IG (*r* = 0.30) and CG (*r* = 0.38) showed a medium effect.

## Discussion

The aim of the present study, the first of its kind, was to test via a brief group intervention on physical activity the applicability of the HAPA model in an acute cardiac ward setting. In contrast to previous studies (i.e. Scholz et al. [16]; Sniehotta et al. [25]; Ziegelmann [11]), this study is not based on or built within a rehabilitation programme or a patient-specific exercise programme but rather one that actively encourages the integration of physical activity in everyday life. Also, the present study did not focus on a specific prescribed exercise plan; rather, patients were invited to self-select the kind of physical activity they preferred. In the IG, only general recommendations concerning heart healthy physical activity were given for example to favour endurance sports such as swimming, running, cycling, etc.

At the baseline, there was an unexpected significant difference in reported physical activity between IG patients and CG patients. There are a number of potential explanations for this. First, participation in the IG was voluntary. It is possible therefore that the more physically active patients were more attracted by the opportunity to participate in the intervention. Second, it is widely acknowledged that practising sport or being physically active is desirable. Hence, it could be that patients who were already physically active were more willing to participate because they were provided in doing so with an opportunity to give an account of their active lifestyle, whereas inactive cardiac patients may be concerned about getting defamed within the group setting for their inactivity.

While controlling statistically for the above baseline difference in reported physical activity, both groups nonetheless indicated high intentions for physical activity after discharge. In spite of this, the results reveal that reported physical activity for IG patients is significantly higher than CG patients in the first 2 months after discharge (*p* < 0.01).

However, in the long term, that is over 6 months, the revealed difference diminished. A possible explanation for this finding could be that patients not participating in the IG need more time (6 months instead of 2 months) to develop intention, action and coping plans and to initiate the desired health behaviour. In support, IG patients increased their physical activity 4 months earlier than CG patients, possibly due to the detailed plans generated within the IG.

Prior studies revealed that cardiac rehabilitation improves patient self-care and helps them to increase their physical activity [26]. To control for this effect, patients were asked to indicate whether they had participated in any other type of (inpatient or outpatient) cardiac rehabilitation programme. In the CG and in the IG, a similar number of patients (CG: *N* = 11; 14.1 %, IG: *N* = 13; 11.3 % *p* = 0.566) reported that they had completed a (inpatient or outpatient) cardiac rehabilitation programme within the 6-month follow-up period. Therefore, it is unlikely that participation in a cardiac rehabilitation programme accounted for the significant difference in physical activity revealed between CG and IG over the first 2 months.

In general, studies applying the HAPA model revealed significant differences in physical activity change between a CG and an IG (i.e. Scholz and Sniehotta [16]; Scholz et al. [27]). The study reported here also found significant differences at the 2-month follow-up period but not at the 6-month follow-up period.

## Limitations and future research suggestions

A potential limitation to this study has to do with the above-revealed differences in reported physical activity between the CG and IG at baseline. Consequently, future researchers should make special note to ensure that participants are as similar as possible in their profile.

A further and connected potential limitation to this study has to do with the absence of randomisation in the assignment process of patients to each of the CG and IG. It should be noted however that in many clinical settings, complete randomisation may be difficult. In such cases, researchers should take care to adopt an approach that balances an awareness of the implications of non-randomisation with the practical constraints associated with the study context.

Additional suggestions for future research include extending the study to incorporate actual physical activity alongside reported physical activity. This could for example be achieved by providing patients with a pedometer which they would use for a specified period prior to returning it for analysis.

Finally, to stabilize changes in physical activity in the long term, a repeated intervention during a cardiac rehabilitation programme following acute care might also be necessary. In this regard, additional options might include the adaptation of existing interventions enriched through other theoretical perspectives noted for their potential with regard to sustained behaviour change (i.e. self-determination theory of Deci and Ryan [28]). In addition, the integration of modern technology such as the use of mobile health apps as well as other online tools to support the maintenance of new physical behaviours and lifestyle changes could also be considered.

## Conclusions

The present study reveals that a brief health psychological intervention based on the HAPA model can be successfully administered within the daily routine of an acute cardiac ward to induce desired behaviour change. Specifically, the intervention discussed herein was able to increase patient’s intention to become more physically active and to increase their actual physical behaviour with respect to everyday activities in the short term. Further studies are needed to explore maintaining the behaviour over the long term.

### Funding

The present study was funded by the Quality Promotion Program of the Tyrolean Health Fund [Tiroler Gesundheitsfonds; #10 − 02/78] and by the Austrian Heart Fund [Österreichischer Herzfonds].

### Informed consent

Informed consent was obtained from all patients for being included in the study.

## References

[1] WHO Global Health Observatory. http://apps.who.int/ghodata (2011). Accessed 19 April 2011.

[2] Yusuf S, Hawken S, Ounpuu S (2014). Effect of potentially modifiable risk factors associated with myocardial infarction in 52 countries (the INTERHEART study): case-control study. Lancet.

[3] Angermayr L, Melchart D, Linde K (2010). Multifactorial lifestyle interventions in the primary and secondary prevention of cardiovascular disease and type 2 diabetes mellitus-a systematic review of randomized controlled trials. Ann Behav Med.

[4] Conn VS, Hafdahl AR, Moore SM (2009). Meta-analysis of interventions to increase physical activity among cardiac subjects. Int J Cardiol.

[5] Ferrier S, Blanchard CM, Vallis M (2011). Behavioural interventions to increase the physical activity of cardiac patients: a review. Eur J Cardiovasc Prev Rehabil.

[6] Held C, Iqbal R, Lear SA (2012). Physical activity levels, ownership of goods promoting sedentary behavior and risk of myocardial infarction: results of the INTERHEART study. Eur Heart J.

[7] Orbell S, Sheeran P (2000). Motivational and volitional processes in action initiation: a field study of the role of implementation intentions. J Appl Psychol.

[8] Knoll N, Scholz U, Rieckmann N (2005). Einführung in die Gesundheitspsychologie.

[9] Schwarzer R, Schwarzer R (1992). Self-efficacy in the adoption and maintenance of health behaviors: theoretical approaches and a new model. Self-efficacy: thought control of action.

[10] Platter M, Hölzl C, Hofer M (2012). Impulse for heart-healthy diet: A short health psychological intervention at the cardiology ward. J Clin and Exp Cardiolog.

[11] Ziegelmann JP, Lippke S, Merten F (2004). Exercise planning and strategy usage in young, middle-aged, and older rehabilitation patients. J Psychosom Res.

[12] Wiedemann AU, Lippke S, Reuter T (2011). The more the better? The number of plans predicts health behaviour change. Appl Psychol Health Well-Being.

[13] Koring M, Richert J, Lippke S (2012). Synergistic effects of planning and self-efficacy on physical activity. Health Educ Behav.

[14] Sniehotta FF, Scholz U, Schwarzer R (2005). Bridging the intention-behaviour gap: planning, self-efficacy, and action control in the adoption and maintenance of physical exercise. Psychol Health.

[15] Sniehotta F, Schwarzer R, Scholz U (2005). Action planning and coping planning for long-term lifestyle change: theory and assessment. Eur J Soc Psychol.

[16] Scholz U, Sniehotta FF (2006). Langzeiteffekte einer Planungs- und Handlungskontrollintervention auf die körperliche Aktivität von Herzpatienten nach der Rehabilitation. Z Gesundheitspsychol.

[17] Gollwitzer PM, Brandstätter V (1997). Implementation intentions and effective goal pursuit. J Pers Soc Psychol.

[18] Whooley MA, Avins AL, Miranda J, Browner WS (1997). Case-finding instruments for depression: two questions are as good as many. J Gen Intern Med.

[19] Schwarzer R, Jerusalem M, Weinman C, Wright S, Johnston M (1995). Generalized Self-Efficacy scale. Measures in health psychology: a user’s portfolio. Causal and control beliefs.

[20] Schwarzer R. Allgemeine Selbstwirksamkeitserwartung. 2011. http://userpage.fu-berlin.de/~health/germscal.htm. Accessed 29 Oct 2011.

[21] Luszczynska A, Gutiérrez-Dona B, Schwarzer R (2005). General self-efficacy in various domains of human functioning: evidence from five countries. Int J Psychol.

[22] Sniehotta FF, Scholz U, Schwarzer R (2005). Long-term effects of two psychological interventions on physical exercise and self-regulation following coronary rehabilitation. Int J Behav Med.

[23] Dlugosch GE, Krieger W (1995). Fragebogen zur Erfassung des Gesundheitsverhaltens (FEG)-Handanweisung.

[24] Fritz CO, Morris PE, Richler JJ (2012). Effect Size Estimates: current Use, Calculation, and Interpretation. J Exp Psychol Gen.

[25] Sniehotta FF, Scholz U, Schwarzer R (2006). Action plans and coping plans for physical exercise: a longitudinal intervention study in cardiac rehabilitation. Br J Health Psychol.

[26] Chatziefstratiou AA, Giakoumidakis K, Brokalaki H (2013). Cardiac rehabilitation outcomes: modifiable risk factors. Br J Nurs.

[27] Scholz U, Sniehotta FF, Burkert S (2007). Increasing physical exercise levels: age-specific benefits of planning. J Aging Health.

[28] Deci EL, Ryan RM (2000). The “What” and “Why” of Goal Pursuits: human Needs and the Self-Determination of Behavior. Psychol Inq.

